# Contextual and psychosocial factors associated with latrine utilization in rural communities in Ethiopia

**DOI:** 10.3389/fpubh.2024.1387861

**Published:** 2024-12-11

**Authors:** Eyasu Bamlaku Golla, Habtamu Geremew, Alegntaw Abate, Mohammed Ahmed Ali, Mulat Belay Simegn, Smegnew Gichew Wondie, Hawi Kumbi, Samuel Abdisa Kuse

**Affiliations:** ^1^College of Health Sciences, Oda Bultum University, Chiro, Ethiopia; ^2^Department of Medical Laboratory Science, College of Health Science, Oda Bultum University, Chiro, Ethiopia; ^3^Department of Midwifery, College of Health Science, Oda Bultum University, Chiro, Ethiopia; ^4^Department of Public Health, College of Medicine and Health Science, Debre Markos University, Debre Markos, Ethiopia; ^5^Department of Human Nutrition, College of Medicine and Health Science, Mizan Tepi University, Mizan Aman, Ethiopia; ^6^Department of Laboratory, Adama Hospital Medical College, Adama, Ethiopia

**Keywords:** latrine utilization, contextual factors, psychosocial factors, wealth index, rural Ethiopia

## Abstract

**Background:**

Although proper latrine utilization is one of the best ways to reduce the risk of infection, it remains a challenge in the majority of rural communities in developing countries such as Ethiopia. Studies have demonstrated the link between individual behavior and latrine use, but there is a paucity of evidence on individual risk perception, perceived social pressure norms, social identity, and perceived ability, which plays an indubitable role in health and behavior change, especially in rural communities.

**Objective:**

This study aimed to identify contextual and psychosocial factors associated with latrine utilization among rural communities in Lomabosa district, Ethiopia.

**Methods:**

A rural community-based cross-sectional study was conducted in June 2022 among rural households (HHs) in Lomabosa district, Ethiopia. A systematic random sampling method was used to select participant households. Data were collected using a pretested structured questionnaire via face-to-face interviews and on-the-spot observations. Stata version 14.1 software was used for statistical analysis. A binary logistic regression model was used to run the bivariable and multivariable analysis of the data. Variables with *p* < 0.25 at bivariable logistic regression analysis were entered into the multivariable analysis. The adjusted odds ratio (AOR) with a 95% confidence interval (CI) was used to show the strength of the association, and the statistical significance was declared at *p* < 0.05.

**Results:**

Of the 682 computed sample sizes, 665 households participated in this study with a response rate of 97%. Accordingly, our analysis found that 67% (95% CI, 63.7–70.5) of households utilize their latrine properly. Educational status (AOR = 2.01; 95% CI: 1.01–2.08), wealth index (AOR = 2.3; 95% CI: 1.23–3.58), perceived susceptibility (AOR 3.2; 95% CI: 1.26–5.14), injunctive norm (AOR 1.9; 95% CI: 1.13–3.18), and perceived ability (AOR 1.9; 95% CI: 1.04–3.79) were identified as contextual and psychosocial factors associated with latrine utilization.

**Conclusion and recommendations:**

This study found that educational status, wealth index, perceived susceptibility, injunctive norm, and perceived ability were the contextual and psychosocial factors associated with latrine utilization. Therefore, information intervention for the low perception of health risk, persuasive and normative interventions for changing norm factors, and infrastructural and other ability support for ability factors should be addressed.

## Introduction

Latrine is a tool for disposing of human excreta safely to ensure a clean and healthy living environment and to prevent communicable diseases caused by excreta. In addition, it “is the lowest cost option to provide privacy and dignity” (1, 2).

Billions of people worldwide lack access to properly managed sanitation facilities. According to the WHO Joint Monitoring Program (JMP) Report in 2020, 494 million people practice open defecation in fields, waterways, and open trenches, without proper waste disposal. The majority of these individuals are from sub-Saharan Africa (18%) and Central and Southern Asia (12%) (3).

In the African context, an open defecation reduction performance report indicated that open defecation has increased rather than decreased. This is because sanitation activities are unable to keep pace with the population growth rate, and some open-defecation-free communities slipped back to open defecation. This makes it challenging to achieve sustainable development goals (SDG), particularly Goal 6 targeted to “end open defecation” by 2030. As a result, in Africa, it is estimated that 1.8 million people die annually due to diarrheal disease, and more than 80% of them are children under the age of 5 years. Therefore, people are at risk of sanitation-related infection, where the majority of people dispose of human excreta in an unsafe way; thus, it needs to be brought to an end (4, 5).

Access to water supply and sanitation in Ethiopia is among the lowest in sub-Saharan Africa. In Ethiopia, only 10% of rural households (HHs) fulfill the requirement for improved latrine facilities, which in turn do not protect against the spread of communicable diseases. Approximately 80% of the disease burden in Ethiopia is related to poor sanitation and hygiene (6). In Ethiopia, a National Strategy for Improved Hygiene and Sanitation has been developed, emphasizing that “on-site” hygiene and sanitation should be managed at the household level with direct support from health extension workers and community-level resources. The focus is on “using local resources more effectively” to increase access to and use of latrines while also encouraging attitudinal changes that lead to improved sanitation and hygiene practices. Understanding the technical options that people want, can afford, and are willing to use was a central pillar of the strategy (7, 8).

To address this, Ethiopia’s Ministry of Health, along with various non-governmental organizations (NGOs), is working together to ensure 100% sanitation coverage through different interventional approaches, such as health extension programs, the Health Development Army, and the Community-Led Total Sanitation and Hygiene (CLTSH). As a result, latrine coverage has shown good progress (73%) (8). Despite this, 27% of households with access to latrines defecated in the open, indicating that access to latrines does not imply latrine use, as many individuals who own latrines do not consistently use them (9). This creates challenges for many districts in Ethiopia, including the Lomabosa district, to achieve the SDG goals on sanitation (9, 10).

Few recent studies in Ethiopia have shown links between contextual factors and psychosocial factors, suggesting that these factors can influence changes in health behavior in practicing sanitation, particularly latrine utilization (11).

Psychosocial factors influence an individual’s psychological and/or social well-being in their social environment. Risk perception (a person’s understanding and awareness of health risk), perceived social pressure norms, social identity, and perceived ability were the psychosocial factors influencing latrine utilization, which plays an indubitable role in health and behavior change (11–13).

Contextual factors are factors related to the individual setting and/or environment that can influence the use of a latrine. These include socioeconomic and demographic characteristics of the household. They may alter the psychosocial factors’ influence on behavior; for instance, a person might be strongly committed to using a latrine, but the commitment may not translate into behavior due to low income. A person with a low income might perceive the materials needed to construct latrines as expensive, while a person with a high income may perceive it as affordable (14).

To address the above factors, several conceptual frameworks have been drawn from various behavioral theories. The risk, attitude, norm, ability, and self-regulation (RANAS) systematic behavioral change approach was one of those approaches to design specific intervention strategies for specific factors: information interventions to address low perceptions of health risk, normative interventions to change social norms, infrastructural and support interventions to improve ability factors, planning and relapse prevention interventions for self-regulation factors—an area that was overlooked in previous studies (14, 15). Therefore, this study aimed to identify the contextual and psychosocial factors associated with latrine utilization among rural communities in the Lomabosa district, Southern Ethiopia. The findings of this study could provide valuable information to local stakeholders, health professionals, and NGOs for designing intervention programs, specifically targeting the psychosocial and contextual factors identified. This, in turn, will add to the existing body of knowledge and play a key role in reducing the spread of communicable diseases.

## Materials and methods

### Study setting

Lomabosa (also known as Loma) was one of the 77 woredas in the South West Region of Ethiopia. The district is 478 km far from Addis Ababa, the capital city of Ethiopia. According to the 2018 CSA population forecast, the district has a total population of 112,953 of these, 97,345 were rural dwellers. It has 2 urban and 24 rural kebeles (small administrative units). Agriculture is the primary livelihood for more than 85% of the population in these rural areas. According to the Loma District Health Office report in 2022, latrine coverage was 92%. The study was conducted in the Lomabosa district because, despite the reports of high levels of latrine coverage, fecal–oral diseases remain prevalent. Common conditions among adults include typhoid fever, bacillary dysentery, helminthiasis, and giardiasis. Diarrhea continues to be one of the leading causes of morbidity in children under 5 years, and cholera was reported as an outbreak in 2020. These issues are either directly or indirectly related to water and sanitation (16).

### Study design and period

For this study, we employed a community-based cross-sectional study design in June 2022, focusing on the rural community in the Lomabosa district.

### Population

All rural households in the Lomabosa district were considered as the source population for our study. The study populations were all rural households in randomly selected kebeles of Lomabosa district.

### Eligibility criteria

Respondents who were older than 18 years and had lived in the study area for more than six months prior to data collection were included in the study while respondents who were unable to respond due to mental disorders, those who temporary replaced the household for taking care of the household were excluded from the study.

### Sample size determination

Epi-info version 7 Info statistical software was used to determine the sample size with the assumption that the proportion of *p*-value from the previous study was 71% (15), 95% CI, 80% power of the test, design effect of 2, and 10% non-response rate, then 682 households were recruited. The sample size for associated factors was also estimated using the double population proportion formula. Finally, to achieve the overall objectives of this study, the larger sample size, that is, 682 was taken as our final sample size.

### Sampling technique

**Figure 1:**
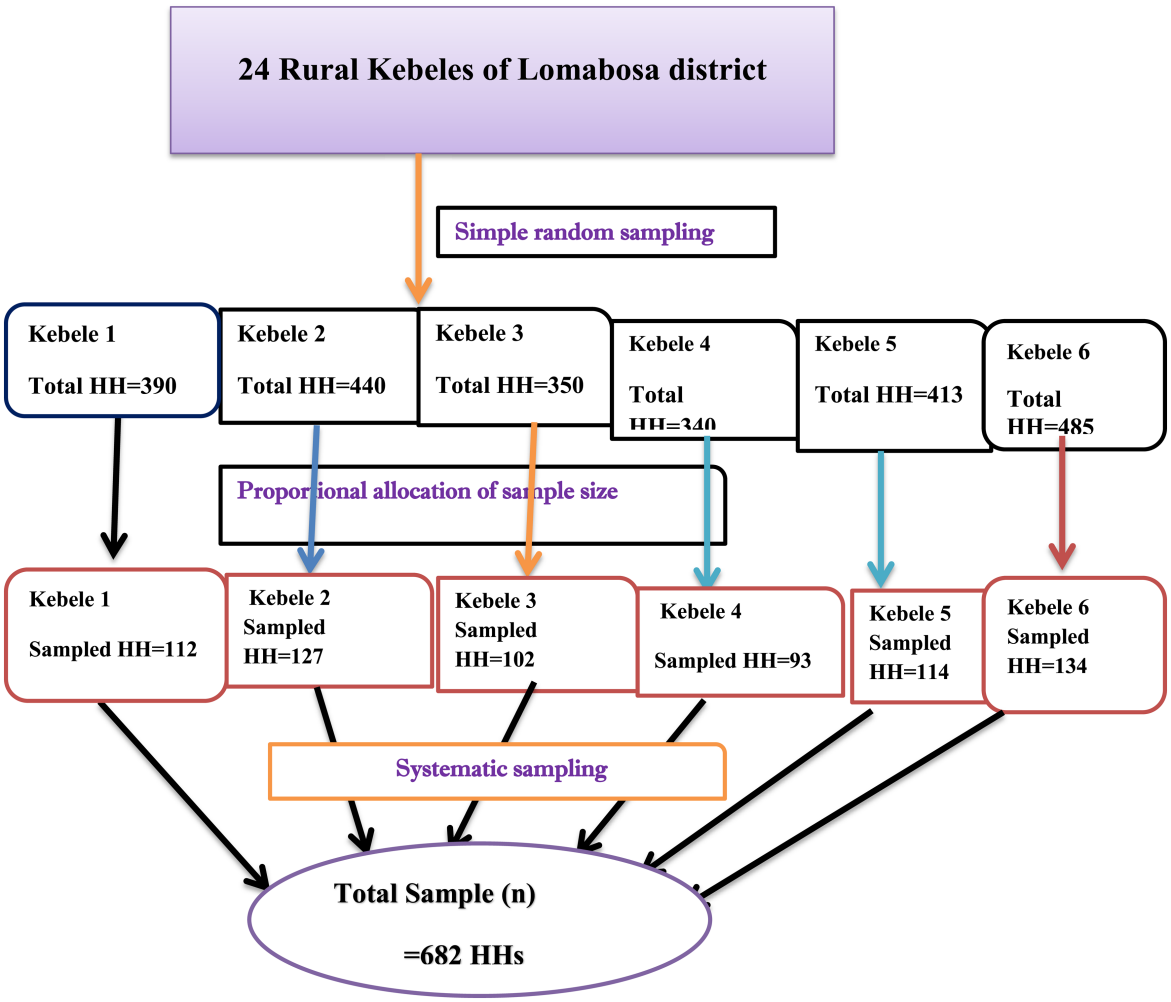
Sampling procedure among rural communities in Lomabosa district, southern Ethiopia, 2022.

A multistage systematic sampling technique was employed to reach the study participants. From the districts’ first 24 rural kebeles (small administrative units), 6 kebeles were randomly selected by taking the name and list of all kebeles as a sampling frame. After that, the sample size was allocated proportionally to the size of households for each selected kebeles. Then, the interval (*K*th) was calculated by dividing the number of households with the sample size allocated for each kebeles (*k* = 3). After the 𝐾th-value was determined as an interval, the study households were systematically selected after randomly selecting the first household and continued by 𝐾th 𝑎𝑛 interval that was calculated (Figure 1).

### Variables and measurements

The outcome variable for this study was latrine utilization, which has binary outcomes as “Yes (1)” and “No (0).” Latrine utilization was cheeked from observation of latrine use parameter (17, 18).

The wealth index was generated using principal components analysis (PCA) from the household’s ownership of selected assets, such as television, the material used for construction, water access, and sanitation facilities. Finally, we categorized them as rich, medium, and poor (16).

Psychosocial variables were assessed using the RANAS behavioral model with a 5-point Likert scale ranging from “strongly disagree” to “strongly agree” (1–5). Its detail is summarized, and each measurement is described in Table 1. A single Likert scale question that ranges from 1 to 5 was used to assess perceived susceptibility, perceived severity, social dilemma, and social identity. The responses of 1–3 were recorded as low, whereas a response of 4–5 was recorded as high (14). Descriptive norm, injunctive norm. Attitude and perceived ability were assessed using a three-item Likert scale question. The score ranged from 3 to 10 was recorded as low, and a score greater than 10 was recorded as high. All variables were coded so that high values were favorable to the behavior (14, 15, 18).

**Table 1:** Measurements that were used to assess the psychosocial predictor’s latrine use among rural communities in Lomabosa district, Ethiopia, 2022 (*n* = 665).

Factors	Items	Responses	Value
Risk perception			
Susceptibility Severity	1. How high /low are the chances that you contract diarrheal disease when defecating in the open field? 2. If you have diarrheal disease because of open defecation, how severely would that impact your life?	Five-point scale, that ranges from almost Very low, to Very high for each question	1–5
Attitude	1.How much beneficial/important it is to defecate using latrine regularly 2. How much do you like to use latrine? 3. How much do you do you enjoy defecating in latrine?	Five-point scale, that ranges from almost Very low, to Very high for each question	1–5
Descriptive norm perception	1.Most of the people I know in the community defecate using latrine regularly 2. Many of your neighbors use latrine for defecation 3.Using latrine regularly is the right thing to do because everybody does so	Five-point scale for each question	
Injective norm perception	1.people who are important to you approve /disapprove that you use latrine 2. Defecating using latrine regularly is something that most of the people in my village think 3.People in my village will judge me if I defecate in the open field	Five-point scale, that ranges from completely disagree to completely agree for each question	1–5
Ability	1.You are confident in your ability to use latrine regularly 2.You are confident that you can maintain your latrine if broken 3. You are confident in your ability to restart using the latrine for defecation even after it was broken for several weeks.”	Five-point scale	1–5
Social identity	I have a lot in common with other community members in terms of latrine utilization	Five-point scale, that ranges from completely disagree to completely agree	1–5
Social dilemma	Community in your village is intensely working together in improving local sanitation	Five-point scale, that ranges from completely disagree to completely agree	1–5

### Data collection procedures

A structured questionnaire and observational checklist were developed after reviewing relevant literature (11, 12, 15, 17, 18). The questions to assess psychosocial variables were adapted from RANAS and from other behavioral studies that applied the same model by which its applicability was confirmed in many previous studies (12–15, 18). Then four data collectors who have a smartphone (Android) were recruited to collect the data through face-to-face interviews with the household head or the housewife using a prepared tool by open data kit (ODK) data collection, and latrine utilization was verified through observation of at least two signs from a sign of latrine use (17, 19). Two supervisors guided the data collectors.

### Data quality control

The questionnaire was written in English, then translated into local language and back to English to ensure the translation accurately represented the original meaning. Before data collection, the questionnaire was pretested in a similar setting on 5% of the sample size in nearby non-selected kebele. To check the internal reliability of the Likert scale question, Cronbach’s *α* was computed and was accepted and cited sequentially (*α* = 0.82). Two days of training for data collectors and supervisors were given. The precoded skip patterns, data types, ranges, and restrictions in ODK collection greatly helped maintain the data quality and reduce errors throughout the data collection period. Similarly, ODK collection has helped to control the daily data collection process remotely. Incorrectly filled data were identified daily, and the correction was performed by respective data collectors. A close monitoring of the whole data collection process was carried out by the supervisors.

### Data processing and analysis

Data was collected using an electronic data collection method via ODK version 2022.3.3 software and stored on the KoBo Collect humanitarian response website. The collected data were downloaded in Microsoft Excel format. Then downloaded data were imported to Statistical Product and Service Solutions (SPSS) version 26.0 software for data cleaning, recoding, and statistical analysis. Descriptive statistics were used to present the data with the frequency, proportion, and median, while texts, tables, and figures.

Using household assets, livestock, and agricultural land ownership, the wealth index of the household was determined using household assets, livestock, and agricultural land ownership (9), and it was generated through PCA; the wealth index places individual households on a continuous scale of relative wealth. Each household asset was assigned a weight or factor score generated through PCA. The resulting asset scores were standardized to a standard normal distribution with a mean of zero and a standard deviation of one. These standardized scores were then used to create the breakpoints that define the wealth index as poor, medium, and rich. Bivariable logistic regressions were performed to see each independent variable’s crude significant relation with latrine utilization. The multivariable logistic analysis model included variables with a *p* ≤ 0.25 at bivariable logistic analysis. Before the inclusion of factors in the final logistic regression model, multicollinearity was checked among the independent variables by using the variance inflation factor (VIF), and there was no evidence of multicollinearity among the explanatory variables (VIF ≤ 1.54). The model also has a good fit since the Hosmer–Lemeshow test for goodness-of-fit could not reject the hypothesis of the model fitness as *p* = 0.271. The adjusted odds ratio (AOR) and its 95% confidence interval (CI) were used to measure the strength and significance of the association.

## Results

### Sociodemographic characteristics of the respondents

In the study, a total of 665 systematically selected households participated, with a response rate of 97.5%. The majority of the respondents, 589 (88.6%), were from male-headed households. Relating to the family size, 387 (58.2%) households had <5 family members. Approximately 419 (63%) of the households have low income. The demographic characteristics of the respondents are summarized in Table 2.

**Table 2:** Socio-demographic characteristics of the respondents among rural communities in Lomabosa, Ethiopia, 2022 (*n* = 665).

Characteristics		*N* = 665	
		Frequency	Percent (%)
Sex of HH head male Female		589 76	88.6 11.4
Age(year)	18–29 yrs. 30–39 yrs. 40–49 yrs. 50–59 yrs. >60 yrs.	192 219 164 63 27	28.9 32.9 24.7 9.5 4.1
Marital status	Married Never married widowed divorced	604 17 23 21	90.8 2.6 3.5 3.2
Educational status	No formal education Primary (1–8) secondary (9–12) college & above	502 118 33 12	75.5 17.7 5 1.8
Occupation status	Farmer Gov’t employ Non gov’t employed Other	578 31 30	86.9 4.7 4.5 3.9
Family size	< 5 members ≥5 member	387 278	58.2 41.8
Wealth index	poor Medium High	419 194 52	63.1 29.2 7.8

HH, household.

### Observation findings

**Figure 2:**
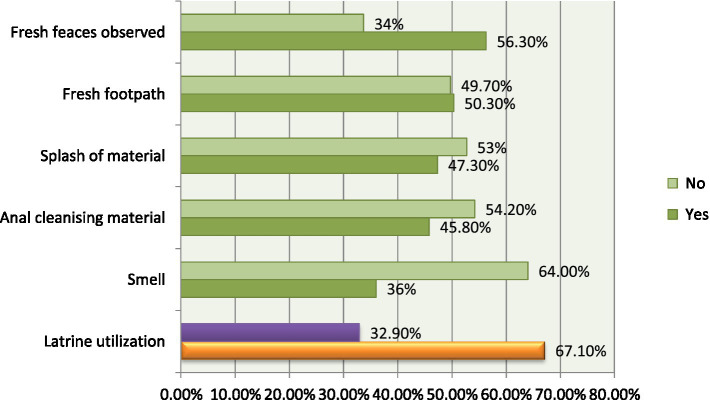
Latrine utilization status through observation, Lomabosa district, Ethiopia, 2022 (*n* = 665).

According to our observation, 446/665 (67.1, 95% CI, 63.71, 70.49%) utilize their latrine properly (Figure 2).

### Psychosocial-related characters of the respondents

According to our findings, approximately 589 out of 665 (88.6%) HHs had High perceived susceptibility toward the chances that they contract the diarrheal disease when defecating in the open field, and also 562 out of 665 (84.5%) HHs had high perceived severity (impact their life if they contract diarrhea). Approximately 458 (69%) HHs were positive toward latrine utilization. Concerning their norms toward latrine utilization, approximately 460 out of 665 (69.2%) HHs had a low perception of how others behave/practice (descriptive norm), and 467 (70.2%) HHs had a low perception of what others expect them to behave/their important referents approved of or disapproved them to use latrines (injective norms) (Table 3).

**Table 3:** Psych-social related characters of the respondents, Lomabosa district, Ethiopia, 2022 (*n* = 665).

Factors		Items	Responses					Categories	
			1	2	3	4	5	Low	High
Perceive susceptibility		How high /low are the chances that you contract diarrheal disease when defecating in the open field	19 (2.9%)	49 (7.4%)	10 (1.5%)	505 (75.9%)	82 (12.3%)	76 (11.4%)	589 (88.6%)
Perceive severity		If you have diarrheal disease because of open defecation, how severely would that impact your life?	27 (4.1%)	35 (5.3%)	39 (5.9%)	419 (63.1%)	145 (21.8%)	103 (15.5%)	562 (84.5%)
Attitude	1	How much beneficial/important it is to defecate using latrine regularly	22 (3.3%)	80 (12%)	26 (3.9%)	417 (62.7%)	120 (18.1%)	207 (31.1%)	458 (68.9%)
	2	How much do you like to use latrine?	38 (5.7%)	119 (17.9%)	30 (4.5%)	360 (54.1%)	118 (17.7%)		
	3	How much do you do you enjoy defecating in latrine?	57 (8.6%)	135 (29.3%)	32 (4.8%)	362 (54.4%)	79 (11.9%)		
Descriptive norm	1	Most of the people I know in the community defecate using latrine regularly	74 (11.1%)	70 (10.5%)	23 (3.5%)	320 (48.1%)	178 (26.85)	205 (30.8%)	460 (69.2%)
	2	Many of your neighbors use latrine for defecation	83 (12.5%)	70 (10.5%)	28 (4.2%)	288 (43.3%)	196 (29.5%)		
	3	Using latrine regularly is the right thing to do because everybody does so	88 (13.2%)	78 (11.7%)	23 (3.5%)	320 (49.1%)	156 (23.5%)		
Injunctive norm	1	People who are important to you approve /disapprove that you use latrine	80 (12%)	83 (12.5%)	36 (5.4%)	291 (43.8%)	175 (26.3%)	198 (29.8%)	467 (70.2%)
	2	Defecating using latrine regularly is something that most of the people in my village think	104 (15.6%)	61 (9.2%)	26 (3.9%)	309 (46.5%)	165 (24.8%)		
	3	People in my village will judge me if I defecate in the open field	35 (5.3%)	76 (11.4%)	77 (11.6%)	282 (42.4%)	195 (29.3%)		
Ability	1	You are confident in your ability to use latrine regularly	78 (10.9%)	77 (11.6%)	35 (5.3%)	298 (44.8%)	182 (27.4%)	202 (30.4%)	463 (69.6%)
	2	You are confident that you can maintain your latrine if broken	80 (12%)	61 (9.2%)	24 (3.6%)	285 (42.9%)	215 (32.3%)		
	3	You are confident in your ability to restart using latrine for defecation even after it was broken for several weeks.”	124 (18.6%)	47 (7.1%)	44 (6.6%)	294 (44.2%)	156 (23.5%)		
Social identity		I have a lot in common with other community members in terms of latrine utilization	66 (9.9%)	138 (20.8%)	35 (5.3%)	281 (42.3%)	145 (21.8%)	241 (36.2%)	424 (63.8%)
Social dilemma		Community in your village is intensely working together in improving local sanitation in latrine utilization	76 (11.4%)	69 (10.4%)	29 (4.4%)	320 (48.1%)	71 (10.7%)	172 (25.8%)	493 (74.2%)

1 = Very low/completely disagree 2 = Low/Disagree 3 = Neither 4 = High/Agree 5 = Very High/Completely Agree.

### Contextual and psychosocial factors associated with larine utilization

The results of the logistic regression showing the crude and adjusted effects of sociodemographic factors and psychosocial factors associated with latrine utilization are summarized in Table 4. On multivariable logistic regressions analysis of the final model, the educational status of the head of the HHs (AOR = 2.01; 95% CI: 1.01–2.08) and wealth index (AOR = 2.3; 95% CI: 1.23–3.58) were sociodemographic factors associated with latrine utilization. Among psychosocial factors, perceived susceptibility (AOR 3.2; 95% CI: 1.26–5.14), injunctive norm (AOR 1.9; 95% CI 1.13–3.18), and perceived ability (AOR 1.9; 95% CI 1.04–3.79) were associated with latrine utilization.

**Table 4:** Bi-variable and Multivariable logistic regression of factors associated with latrine utilization among rural communities in Lomabosa district, Ethiopia, 2022 (*n* = 665).

Variables	Categories	Latrine utilization	COR (95%CI)	AOR (95%CI)
		Yes (%) No (%)		
Sex of household head Educational status	Male Female Attend formal education Not attend	396(67.6) 193(32.4) 50(65.8) 26(34.2) 134(82.2) 29(17.8) 314(62.6) 188(37.4)	1.1(0.65,1.79) 1 2.8(1.78,4.29) 1	1.1(044, 1.3) 1 **2.01(1.01,2.08)** * 1
Family size	< 5member ≥5 member	289(74.7) 98(25.3) 159(57.2) 119(42.8)	2.2(1.59,3.07) 1	1.07(0.532.18) 1
Occupational status	Farmer Housewife Other	355(66.2) 181(33.8) 27(81.8) 6(18.2) 66(68.7) 30(31.3)	1.2(0.73,1.79) 1.1(0.68,1.31) 1	1.1(0.70,2.11) 1.1(0.58,1.93) 1
Wealth index	Rich Medium Poor	44(86.6) 8(13.4) 131(67.5) 63(32.5) 271(64.6) 148(35.4)	2.7(1.59,3.07) 1.2(0.81,1.56) 1	2.3(1.23.3.58)* 1.1(0.77,2.22)
Perceived susceptibility	High Low	405(69) 182(31) 43(55.1) 35(44.6)	1.8(1.12,2.92) 1	**3.2(1.26,5.14)** * 1
Perceived severity Attitude	High Low Positive Negative	392(69.8) 170(30.2) 56(54.4) 47(45.6) 315(74.8) 106(25.2) 133(54.5) 111(45.5)	1.9(1.26,2.97) 1 2.5(1.78,3.47) 1	1.35(0.74,2.46) 1 1.7(0.94,2.94) 1
Descriptive norm	High Low	357(77.6) 103(22.4) 91(44.4) 114(55.6)	4.3(3.05,6.18) 1	1.04(0.61,1.79) 1
Injective Norm	High Low	360(77.1) 107(22.5) 89(44.1) 113(55.9)	4.4(3.08,6.24) 1	1.9(1.13,3.18) * 1
Perceived ability	High Low	359(77.5) 104(18.7) 213(56.6) 163(43.4)	3.3(2.33,4.77) 1	1.9(1.04,3.79) * 1
Social identity	High Low	201(68.8) 91(31.2) 247(66.2) 126(33.8)	1.1(0.81,1.56) 1	1.2(0.63,2.33) 1
Social dilemma	High Low	337(71.8) 132(28.2) 111(56.6) 85(43.4)	2.0(1.38,2.77) 1	1.2(0.52,2.62) 1

*p < 0.05. HH, Households, AOR adjusted odds ratio, CI confidence interval, COR crude odds ratio, CLTSH community lid total sanitation. Bold values *means significantly associated at *p* < 0.05.

## Discussion

This community-based cross-sectional study has attempted to identify the psychosocial predictors of latrine utilization in the rural communities of Loma district, Southwest Ethiopia. According to our observation, 67% of the households utilize their latrine properly. This finding was consistent with the study performed in Gurage Zone, Ethiopia (65.8%), and Sebeta district, Oromia, Ethiopia (68%) (20, 21). However, our findings were lower when compared to studies conducted in Hotesa district, Arsi, Ethiopia (81%); Wondo Genet district, South Ethiopia (83%); East Meskan District, Southern Ethiopia (73.3%); and Nepal (94.3%) (19, 22–24). This difference might be in the study period, and our study was conducted in rural areas. In contrast, some were performed in both rural and urban areas, and it is known that the awareness of latrine utilization among urban residents is better than that of rural residents. On the contrary, it might be due to the difference in implementing the health extension package. Our finding showed that latrine utilization remains far below the WHO and Ethiopian WASH plan (8), which needs urgent attention.

In this study, households headed by individuals who had attended formal education were approximately two times more likely to utilize their latrines than the latrines of their counterparts. The finding of this study is supported by other similar studies conducted in Ethiopia concerning HH latrine utilization and its association with the educational status of household heads; it concluded that the utilization level has a significant association with the educational status of household heads (23, 25, 26) in Ethiopia. This might be because education greatly impacts how people behave when engaging in healthy behaviors. Similarly, as educational status increases, knowledge on disposing of human excreta safely to ensure a clean and healthy living environment and prevent communicable diseases caused by excreta increases. As a result, they utilize their latrine properly to keep their health.

The contextual factors indicated that the model explained for about 48% of the overall variability in the results (*p* < 0.05). Adding the psychosocial factors to the regression model resulted in a significant 76.2% increase in explained variation in latrine utilization.

In this final model, the wealth index was the only significant contextual factor. We found that respondents from wealthier households in the district were 2.3 more likely to use their latrines. This might result because as a household’s wealth rises, they can afford the material needed to construct the latrine facilities, so the option to utilize them increases. This finding is supported by the Ethiopian Demography and Health Survey 2019, which found that latrine owners consistently mentioned cost as a barrier to building and upgrading facilities (9, 10).

Regarding psychosocial factors, this study found that perceived susceptibility, injunctive norm, and perceived ability were predictors of latrine utilization. Participants who perceived susceptibility to diarrheal disease due to contamination from not utilizing the latrine properly were approximately 3.2 times more likely to utilize their latrine. The odds of larine use among participants who perceived latrine use behaviors are typically approved or disapproved by referents (Injunctive norm) were 2 times higher than their counterparts. In addition, participants who had high confidence in their ability to practice latrine utilization (perceived ability) were approximately two times more likely to utilize their latrines. This showed that the social norm influenced people’s decision to use a latrine. This study is consistent with the study performed at Dirashe and Becho districts (12, 13, 15); Ethiopia showed that perceptions of minimal health threat from not utilizing a latrine and perceived ability to maintain their latrine influenced latrine utilization. This study was also supported by the study performed in Northern Ghana (27) and Zambia, which found that individuals practice open defecation due to societal norms (28). Given this finding, we believe that normative and persuasive intervention is appropriate for the current setting.

### Limitations of the study

Although this study provided information about contextual and psychosocial variables affecting latrine utilization, the findings should be interpreted with limitations in mind. First, the study was a cross-sectional study, so causality relationships could not be determined. Second, although we acknowledged the strong relationship between water and sanitation, and households’ health, our research did not discuss this relationship because the whole study was conceptualized on latrine utilization. Third, all psychosocial determinants were self-reported which may be biased in reporting their behaviors.

## Conclusion and recommendations

Our results are consistent with behavior change theories and health promotion approaches that stress the importance of contextual and psychosocial factors in enabling or deterring the desired behavior (14, 29, 30). Our results suggest that different factors are associated with households’ latrine utilization. An individual’s decision and action to use a latrine facility is affected by the interplay of psychosocial and contextual factors. According to our findings, educational status, wealth index, perceived susceptibility, injunctive norm, and perceived ability were contextual and psychosocial factors associated with latrine utilization. Therefore, health extension workers, health professionals, district health offices, and local administrators should have undertaken information intervention for the low perception of health risk, persuasive and normative interventions for changing norm factors, and infrastructural and other ability support for ability factors. Messages also need to be integrated within the existing community structures to increase latrine utilization among rural communities. We also recommend future research on contextual and psychosocial factors, particularly qualitative studies, to explore in-depth information to promote behavior change.

## Data Availability

The original contributions presented in the study are included in the article/supplementary material, further inquiries can be directed to the corresponding author.
